# Apolipoprotein A-1 does not appear to be a suitable acute-phase reaction marker in canine babesiosis and hemoplasmosis

**DOI:** 10.1016/j.crpvbd.2025.100340

**Published:** 2025-11-29

**Authors:** Imke M. von Hohnhorst, Andreas Moritz, Clara M. Eisenecker, Christina Strube, Torsten J. Naucke, Elisabeth Müller, Ingo Schäfer

**Affiliations:** a Clinical Pathology and Clinical Laboratory Diagnostics, Department of Veterinary Medicine, Justus-Liebig-University Giessen, Frankfurter Strasse 114, 35392, Giessen, Hesse, Germany; b Small Animal Clinic – Internal Medicine, Department of Veterinary Medicine, Justus-Liebig-University Giessen, Frankfurter Strasse 114, 35392, Giessen, Hesse, Germany; c Institute for Parasitology, Centre for Infection Medicine, University of Veterinary Medicine Hannover, Buenteweg 17, Building 217, 30559, Hanover, Lower Saxony, Germany; d Laboklin GmbH & Co. KG, Steubenstrasse 4, 97688, Bad Kissingen, Bavaria, Germany

**Keywords:** *Babesia canis*, Lipoproteins, Acute-phase protein, Hemotrophic *Mycoplasma*

## Abstract

Acute *Babesia canis* infections in dogs most often cause a marked acute-phase response (APR) influencing lipoprotein metabolism. However, the significance of apolipoprotein A-1 (ApoA-1), a major protein and key determinant of high-density lipoprotein (HDL) formation, as a negative acute-phase protein is unclear. To investigate this topic, 84 dogs were classified in four study groups (SGs): (i) 23 dogs with acute *B. canis* infection tested PCR-positive; (ii) 26 dogs with high antibody levels against *Babesia* spp.; (iii) 17 dogs with acute hemotrophic *Mycoplasma* infections; and (iv) 18 clinically healthy dogs. Complete blood count, total protein, albumin, globulins, triglycerides, cholesterol, and C-reactive protein (CRP) were compared between the SGs and correlation analysis was performed. Results showed that ApoA-1 concentrations did not differ significantly between SGs but were decreased in SG I compared to SGs II-IV. Significantly different concentrations of CRP and triglycerides indicated APR in dogs with acute *B. canis* infection. ApoA-1 concentrations showed a moderate negative correlation with cholesterol and a moderate positive correlation with eosinophils. No statistical significance was found when comparing dogs tested serologically positive for *Babesia* spp., dogs with hemotrophic *Mycoplasma* infections, and clinically healthy dogs. Overall, APR was observed in dogs with acute *B. canis* infections with significantly elevated triglycerides and CRP, but the value of ApoA-1 as a negative acute-phase protein is questionable. No evidence for APR was observed in *Babesia* spp.-seropositive dogs and those with hemotrophic *Mycoplasma* infection.

## Highlights

• Dogs with acute *Babesia canis* infection showed acute-phase reaction (ARP).
• C-reactive protein (CRP) and triglycerides were significantly elevated.
• The value of apolipoprotein A-1 as a negative acute-phase protein was questionable.
• No acute-phase reaction was detected in *Babesia-*seropositive dogs.
• No acute-phase reaction was registered in dogs infected with hemotrophic *Mycoplasma*.

## Graphical abstract

**Figure:**
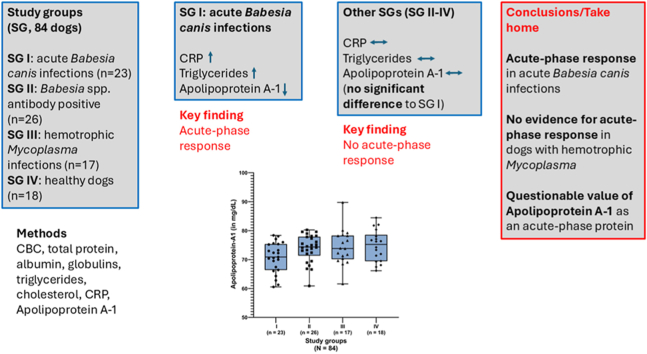

## 1 Introduction

Apolipoprotein A-1 (ApoA-1) is one of the major structural proteins of plasma high-density lipoproteins (HDL). Primary functions of HDL include the mediation of cholesterol homeostasis and the modulation of inflammatory responses in sepsis by binding circulating lipopolysaccharides (LPS) and inactivating bacterial endotoxins (Catapano et al., 2014; Tanaka et al., 2020). In humans, low ApoA-1 concentrations were linked to increased mortality, prolonged intensive care hospitalization, and hospital-acquired infections (Chien et al., 2005; Bermudes et al., 2018). Data in dogs are limited, but ApoA-1 is considered as a potential biomarker of canine systemic inflammatory response syndrome (SIRS) and sepsis acting as a negative acute-phase protein (APP) (Giunti et al., 2015; Escribano et al., 2016). SIRS was recognized in over one-third out of 49 dogs with acute autochthonous *Babesia canis* infections in Germany (Weingart et al., 2023). However, the pathophysiology of decrease in ApoA-1 concentrations accompanied by an infection/inflammation is still unclear and most likely multifactorial (Bulgarelli et al., 2023). A recent study found ApoA-1 as a useful biomarker of sepsis severity in dogs with decrease in septic shock and multiorgan dysfunction syndrome (Bulgarelli et al., 2023).

A marked acute-phase reaction (APR) including elevation of APPs is reported in dogs with acute *B. canis* infections (Seibert et al., 2022; Weingart et al., 2023; Von Hohnhorst et al., 2025), e.g. based on elevated Serum amyloid A (SAA) (Milanovic et al., 2019) and C-reactive protein (CRP) concentrations (Von Hohnhorst et al., 2025). Mortality rates vary from 1 to 20%, with regional impact including highest rates of 12–20% in central and northeastern Europe and lowest rates of < 5% in southwestern Europe (Goddard et al., 2013; Carcy et al., 2015; Turna et al., 2022; Weingart et al., 2023; Eisenecker et al., under review). In 29 dogs with acute *B. canis* infections, ApoA-1 concentrations were increased in positive correlation with SAA compared to 10 healthy dogs. Significantly decreased ApoA-1 concentrations were seen in five dogs with leishmaniasis, as another protozoal vector-borne disease, with a significant increase if treatment was successful (Escribano et al., 2016). ApoA-1 was suggested as a potentially valuable biomarker for monitoring treatment success in canine leishmaniasis (Escribano et al., 2016).

*Myoplasma hemocanis* infections may be widespread but dogs with acute infections most often show a latent course of disease (Messick, 2004). Infections usually only result in clinical signs and hemolytic anemia in splenectomized and/or immunocompromised dogs (Tasker, 2022) and may result in a chronic carrier status after clinical signs resolved (Messick, 2004).

Hypotheses in our study include the presence of an APR in both canine infectious diseases, babesiosis and hemoplasmosis, accompanied by a significant increase in CRP and significant changes in ApoA-1 compared to clinically healthy dogs and dogs tested serologically positive for *Babesia* spp., indicating pathogen contact in the past. A significant difference is especially expected in comparing dogs with fever to dogs without. This study aimed to assess the potential of ApoA-1, CRP, cholesterol, and triglycerides as potential biomarkers in dogs with acute *B. canis* and hemotrophic *Mycoplasma* infections to classify an APR.

## 2 Materials and methods

### 2.1 Study population

This study included 84 dogs, of which EDTA and serum samples were submitted by German veterinarians to the Laboklin laboratory (Bad Kissingen, Germany) from April to December 2024. Questionnaires were sent to the veterinarians asking for stays abroad, clinical signs, rectal temperature, and comorbidities. All dogs tested serologically negative for *Ehrlichia canis* (*Ehrlichia* ELISA Dog, Afosa, Blankenfelde-Mahlow, Germany) and *Leishmania infantum* (NovaTec VetLine *Leishmania* ELISA, Immundiagnostica GmbH, Dietzenbach, Germany), PCR-negative for *Hepatozoon canis* (real-time PCR, target: *16S* rDNA), microfilaria (real-time PCR, target: *16S* rDNA), *Anaplasma phagocytophilum* (real-time PCR, target: *hsp60* gene), and *Anaplasma platys* (real-time PCR, target: *groEL* gene) from EDTA blood, and negative for *Dirofilaria immitis* in antigen ELISA-testing (FASTest® HW Antigen, MegaCor GmbH, Hörbranz, Austria) from EDTA blood. Additionally, dogs were tested by PCR for *B. canis* (forward primer: 5′-AAT ACC CAA TCC TGA CAC AGG G-3’; reverse primer: 5′-TTA AAT ACG AAT GCC CCC AAC-3′), based on Olmeda et al. (1997) (species differentiation by Sanger sequencing) and hemotrophic *Mycoplasma* spp. (*M. hemocanis*, *M. hematoparvum*; real-time PCR, target: *16S* rDNA, based on Barker et al. (2010) from EDTA blood), as well as for *Babesia* spp. antibodies (*Babesia* ELISA Dog, Afosa, Blankenfelde-Mahlow, Germany) in serum. No significant underlying other diseases were reported.

**Fig. 1:**
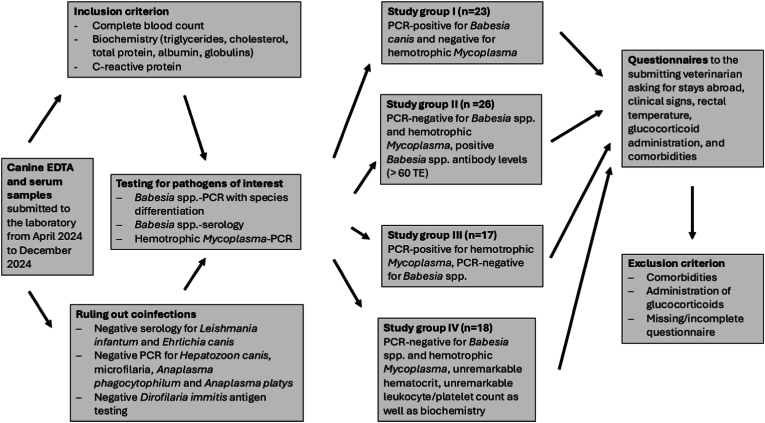
Study design including 84 dogs with determination of Apolipoprotein A-1 concentrations.

According to the PCR results for *B. canis* and hemotrophic *Mycoplasma* as well as serological results for *Babesia* spp., dogs were divided into four different study groups (SGs) (Fig. 1): (i) SG I: dogs with acute babesiosis tested *B. canis*-positive by PCR, but negative in all other mentioned diagnostic tests; (ii) SG II: dogs tested serologically positive for *Babesia* spp. (> 60 technical units (TE) in ELISA-testing), but PCR-negative for *B. canis* and hemotrophic *Mycoplasma*; (iii) SG III: dogs tested PCR-positive for hemotrophic *Mycoplasma* and serologically negative/positive for *Babesia* spp., but negative in all other mentioned diagnostic tests; and (iv) SG IV: dogs classified as clinically healthy based on negative PCR and serology in all mentioned diagnostic tests, absence of hematological abnormalities regarding hematocrit as well as leukocyte and platelet count, absence of any clinical signs, and rectal temperature ≤ 39.0 °C. Samples of dogs classified as clinically healthy were taken from blood donors or routine checkups.

In dogs with acute *B. canis* infection (SG I), additional pathogen quantification was performed using a droplet digital PCR (ddPCR, Bio-Rad) targeting the *B. canis-*specific *Bc28.1* gene. No corticosteroids were applied to any dog included in the study.

### 2.2 Hematological and biochemical analysis including CRP and ApoA-1 measurement

A complete blood count (CBC, Sysmex XN-V analyzer, Sysmex Deutschland, Norderstedt, Germany), measurements of triglycerides, cholesterol, total protein, albumin, and globulins (Cobas 8000, Roche Deutschland Holding GmbH, Mannheim, Germany), and a serum protein capillary electrophoresis test (Sebia Minicap, Sebia, Mainz, Germany) were performed at the Laboklin laboratory (Bad Kissingen, Germany) immediately after arrival of samples, which was no later than 24 h after blood collection. Each thrombocytopenia < 90 G/l was confirmed microscopically.

CRP as an indicator of APR was measured in serum (Cobas 8000, Roche Deutschland Holding GmbH, Mannheim, Germany) at the Laboklin laboratory (Bad Kissingen, Germany) immediately after receipt of samples in the laboratory, including a shipment of a maximum duration of 24 h between blood sampling and analysis.

For measurement of ApoA-1 concentrations, aliquots were performed out of the serum samples immediately after arrival in the laboratory, including a shipment of a maximum duration of 24 h. Aliquots were stored for a maximum of 9 months at −80 °C and did not undergo any freeze-thaw cycle before measurement of ApoA-1 concentrations. ApoA-1 was measured in one day by the immunoturbidimetric Apolipoprotein A1 FS∗ assay with photometric determination of antigen-antibody reactions between antibodies against ApoA-1 and concentration of ApoA-1 in the sample according to the manufacturer’s guidelines using the respons®910 analyzer (DiaSys Diagnostic Systems GmbH, Holzheim, Germany) at the BSL BIOSERVICE Scientific Laboratories Munich GmbH (Planegg, Germany). The ApoA-1 immunoassay was set up using calibration and quality control material provided by the manufacturer. The range of ApoA-1 concentrations was quantified as 0.61 mg/dl to 260.0 mg/dl by the manufacturer, validated for use in dogs with intra- (9 sample replicates measured in triplicate on the same working day) and inter-assay (5 replicates evaluated on five following working days from Monday to Friday) coefficients of variation < 5% at the BSL BIOSERVICE laboratory.

### 2.3 Statistical analysis

For statistical analysis, the software SPSS (version 30.0, IBM) was used; *P* ≤ 0.05 was considered statistically significant. Comparison of hematological and biochemical parameters as well as ApoA-1 concentrations between dogs with and without fever was done by the Mann-Whitney U-test. Kruskal-Wallis testing, including Bonferroni correction, was applied to compare the four different study groups (SG I-IV). Spearman correlation analysis was used to determine potential correlations between hematological results, triglycerides, cholesterol, CRP, ApoA-1 concentrations, *Babesia* spp. antibody levels, and results of serum protein electrophoresis. Correlation coefficients (*ρ*) were classified according to Cohen (1988) as weak (*ρ*: 0.1–0.29), moderate (*ρ*: 0.3–0.49), and strong correlation (*ρ* ≥ 0.5).

## 3 Results

### 3.1 Study groups

Eighty-four dogs were included, with 23 dogs (27.4%) classified in SG I (*B. canis* PCR-positive), 26 (31.0%) in SG II (*Babesia* spp. antibody-positive), 17 (20.2%) in SG III (hemotrophic *Mycoplasma* PCR-positive), and 18 (21.4%) in SG IV (clinically healthy dogs). Data regarding sex, age, stays abroad from Germany, and tick attachment are summarized in Table 1. The most remarkable findings in general examination included lethargy in 26 out of 82 dogs (31.7%; SG I: 21/23, 91.3%; SG II: 2/26, 7.7%; SG III: 3/17, 17.6%; SG IV: 0/18), fever defined as rectal temperature > 39.0 °C in 18 out of 84 dogs (21.4%; SG I: 15/23, 65.2%; SG II: 2/26, 7.7%; SG III: 1/17, 5.9%; SG IV: 0/18), and lymphadenopathy in 4 out of 82 dogs (4.9%; SG I: 2/23, 8.7%; SG II: 1/26, 3.8%; SG III: 1/17, 5.9%; SG IV: 0/16). Statistical analysis revealed statistically significant differences between the SGs for rectal temperature (Table 2).

**Table 1:** Sex, age, anamnesis regarding stays abroad from Germany, and tick attachment in the last two weeks reported by the owners in dogs with acute *Babesia canis* infection (SG I), dogs with positive *Babesia* serology (SG II, > 60 TE), dogs with hemotrophic *Mycoplasma* infection (SG III), and clinically healthy control dogs (SG IV).

Parameter	SG I	SG II	SG III	SG IV
*N*	23	26	17	18
Male, *n*/*N* (%)	10/23 (43.5)	13/26 (50.0)	11/17 (64.7)	13/18 (72.2)
Female, *n*/*N* (%)	13/23 (56.5)	13/26 (50.0)	6/17 (35.3)	5/18 (27.8)
Median age in years (Range)	5 (1–14)	4 (1–8)	6 (2–14)	4.5 (1–12)
Stays abroad from Germany, *n*/*N* (%)	7/23 (30.4; I: 2; T: 3; I + T: 2)	25/26 (96.2; I: 25)	15/17 (88.2; I: 14; T: 1)	8/16 (50.0; I: 2; T: 6)^a^
Tick attachment, *n*/*N* (%)	16/23 (69.6)	5/26 (19.2)	1/17 (5.9)	1/16 (6.3)^a^

*Abbreviations*: I, Import; *n*, number of dogs chosen from a whole population of dogs; *N*, total number of dogs in the set from which a selection is made; T, travel; TE, technical units in enzyme-linked immunosorbent assay (ELISA)-testing.

a Data regarding stays abroad from Germany and tick attachment were available in 16 out of 18 dogs (89%) in SG IV.

**Table 2:** Rectal temperature, hematological and biochemical results, serum protein electrophoresis results, and apolipoprotein A-1 concentrations in dogs with acute *Babesia canis* infection (SG I), dogs with positive *Babesia* serology (SG II, > 60 TE), dogs with hemotrophic *Mycoplasma* infection (SG III), and clinically healthy control dogs (SG IV).

Parameter		SG I (*n* = 23)	SG II (*n* = 26)	SG III (*n* = 17)	SG IV (*n* = 18)	RI^a^	*P*^b^
RT (°C)	Median (Range)	39.4 (37.4–40.2)	38.4 (37.3–39.1)	38.4 (37.9–39.2)	38.4 (38.0–39.0)	≤ 39.0	I *vs* II: *P* = 0.004; I *vs* III: *P* = 0.009; I *vs* IV: *P* = 0.004; Others: *P* > 0.05
	IQR	38.7–39.7	38.2–38.7	38.2–38.6	38.1–38.6 (*n* = 16)^c^		
HCT (%)	Median (Range)	34.0 (19.0–57.0)	53.5 (42.0–58.0)	50.5 (36.0–59.0)	50.5 (44.0–57.0)	44.0–52.0	I *vs* II: *P* < 0.001; I *vs* III: *P* < 0.001; I *vs* IV: *P* < 0.001; Others: *P* > 0.05
	IQR	31.0–39.0	49.8–55.0	46.8–53.0	47.0–53.3		
WBC (×10^9^/l)	Median (Range)	4.6 (1.1–13.5)	9.3 (5.4–16.9)	8.3 (5.3–24.2)	9.0 (6.3–11.3)	6.0–12.0	I *vs* II: *P* < 0.001; I *vs* III: *P* = 0.002; I *vs* IV: *P* = 0.001; Others: *P* > 0.05
	IQR	3.4–7.2	8.3–11.8	6.8–11.1	7.9–9.7		
Seg (×10^9^/l)	Median (Range)	3.1 (0.7–9.3)	4.8 (3.0–12.0)	4.5 (2.4–13.1)	5.3 (3.3–8.4)	3.0–9.0	I *vs* II: *P* = 0.001; I *vs* III: *P* = 0.05; I *vs* IV: *P* = 0.005; Others: *P* > 0.05
	IQR	2.4–4.4	4.3–6.9	4.0–6.0	4.3–6.1		
Lym (×10^9^/l)	Median (Range)	0.7 (0.1–2.8)	2.9 (1.4–4.9)	2.6 (0.6–4.5)	2.2 (1.3–3.6)	1.0–3.6	I *vs* II: *P* < 0.001; I *vs* III: *P* < 0.001; I *vs* IV: *P* = 0.001; Others: *P* > 0.05
	IQR	0.3–1.3	2.2–3.6	1.5–3.4	1.9–3.0		
Eos (×10^9^/l)	Median (Range)	0.0 (0.0–0.1)	0.5 (0.1–5.3)	0.6 (0.3–8.0)	0.6 (0.3–3.3)	0.04–0.6	I *vs* II: *P* < 0.001; I *vs* III: *P* < 0.001; I *vs* IV: *P* < 0.001; Others: *P* > 0.05
	IQR	0.0–0.0	0.3–1.0	0.6–1.5	0.4–0.8		
Mono (×10^9^/l)	Median (Range)	0.8 (0.0–3.4)	0.5 (0.1–1.0)	0.5 (0.2–1.0)	0.6 (0.2–0.8)	0.04–0.5	*P* > 0.05
	IQR	0.3–1.3	0.4–0.7	0.3–0.7	0.4–0.6		
PLT (×10^9^/l)	Median (Range)	33 (11–169)	201.5 (133–362)	218.0 (114–420)	219.5 (170–350)	150–500	I *vs* II: *P* < 0.001; I *vs* III: *P* < 0.001; I *vs* IV: *P* < 0.001; Others: *P* > 0.05
	IQR	21–47)	175–270	161–248	186–256		
TP (g/l)	Median (Range)	49.7 (37.9–62.6)	64.2 (57.0–76.9)	63.1 (57.1–75.9)	60.4 (53.5–64.6)	54.0–57.0	I *vs* II: *P* < 0.001; I *vs* III: *P* < 0.001; I *vs* IV: *P* = 0.007; Others: *P* > 0.05
	IQR	44.1–56.3	61.1–67.4	61.2–66.9	57.2–62.9		
Alb (g/l)	Median (Range)	27.6 (20.7–35.7)	39.5 (29.3–42.6)	36.7 (33.2–40.4)	37.5 (35.0–41.2)	25.0–44.0	I *vs* II: *P* < 0.001; I *vs* III: *P* < 0.001; I *vs* IV: *P* < 0.001; Others: *P* > 0.05
	IQR	26.2–31.5	36.8–40.9	34.3–38.4	36.4–38.8		
Glob (g/l)	Median (Range)	22.0 (13.6–34.5)	25.7 (16.9–36.4)	28.2 (21.0–42.4)	23.2 (15.9–26.4)	< 45.0	I *vs* III: *P* = 0.001; III *vs* IV: *P* = 0.005; Others: *P* > 0.05
	IQR	17.9–24.9	22.4–28.1	23.7–29.7	20.4–24.9		
Chol (mmol/l)	Median (Range)	7.1 (2.9–10.3)	5.5 (3.4–9.7)	5.4 (3.4–8.5)	5.2 (3.5–8.4)	3.1–10.1	*P* > 0.05
	IQR	5.0–8.2	4.7–6.6	4.4–6.4	4.7–7.0		
Trigly (mmol/l)	Median (Range)	1.2 (0.5–4.6)	0.7 (0.3–1.2)	0.6 (0.3–1.8)	0.7 (0.3–3.0)	< 3.9	I *vs* II: *P* < 0.001; I *vs* III: *P* = 0.005; I *vs* IV: *P* = 0.02; Others: *P* > 0.05
	IQR	0.9–1.4	0.5–0.9	0.5–1.1	0.6–0.9		
CRP (mg/l)	Median (Range)	137.7 (51.9–292.5)	1.2 (0.1–12.0)	2.1 (0.1–40.1)	1.1 (0.1–8.0)	< 15.0	I *vs* II: *P* < 0.001; I *vs* III: *P* < 0.001; I *vs* IV: *P* < 0.001; Others: *P* > 0.05
	IQR	92.0–213.3	0.5–2.0 (*n* = 25)^c^	0.6–10.2	0.6–2.8		
Alb/Glob	Median (Range)	1.2 (0.7–1.8)	1.1 (0.7–2.0)	1.1 (0.7–1.7)	1.2 (0.4–2.2)	> 0.59	*P* > 0.05
	IQR	1.1–1.4 (*n* = 22)^c^	1.0–1.4	0.8–1.4	1.0–1.7		
EAlb (g/l)	Median (Range)	26.1 (18.0–34.5)	34.5 (23.7–42.9)	33.5 (26.2–40.2)	33.8 (14.2–39.6)	44–66%^d^	I *vs* II: *P* < 0.001; I *vs* III: *P* = 0.001; I *vs* IV: *P* = 0.004; Others: *P* > 0.05
	IQR	24.2–30.3 (*n* = 22)^c^	31.5–37.9	29.5–37.3	30.4–36.7		
Ealpha (g/l)	Median (Range)	7.7 (4.7–12.7)	8.1 (5.4–12.7)	8.2 (6.3–11.9)	9.1 (5.5–41.5)	9–24%^d^	*P* > 0.05
	IQR	5.8–8.9 (*n* = 22)^c^	7.0–9.6	7.8–9.6	6.4–10.1		
Ebeta (g/l)	Median (Range)	9.4 (4.6–18.7)	12.9 (7.9–22.2)	11.6 (8.2–25.4)	10.3 (3.5–14.8)	9–32%^d^	I *vs* II: *P* = 0.03; Others: *P* > 0.05
	IQR	7.3–11.3 (*n* = 22)^c^	9.9–14.7	9.3–14.2	7.9–12.0		
Egamma (g/l)	Median (Range)	4.6 (3.3–9.2)	8.7 (2.7–13.5)	9.3 (5.0–20.3)	7.0 (2.8–18.1)	5–20%^d^	I *vs* II: *P* < 0.001; I *vs* III: *P* < 0.001; Others: *P* > 0.05
	IQR	4.1–6.2 (*n* = 22)^c^	7.1–10.2	8.6–10.6	4.8–9.8		
ApoA-1 (mg/dl)	Median (Range)	70.9 (60.6–78.4)	74.4 (60.9–80.3)	73.8 (61.6–89.7)	75.2 (66.2–84.5)	–	*P* > 0.05
	IQR	66.4–75.4	71.4–77.9	70.2–78.3	69.5–78.6		

*Abbreviations*: Alb, albumin; ApoA-1, apolipoprotein A-1; Chol, cholesterol; CRP, c-reactive protein; Eos, eosinophils; EAlb, albumin-section serum protein electrophoresis; Ealpha, alpha-globulin-section serum protein electrophoresis; Ebeta, beta-globulin-section serum protein electrophoresis; Egamma, gamma-globulin-section serum protein electrophoresis; Glob, globulin; HCT, hematocrit; IQR, interquartile range; Lym, lymphocytes; PLT, platelets; Mono, monocytes; RI, reference interval; Seg, segmented neutrophils; SG, study group; RT, rectal temperature; TP, total protein; Trigly, triglycerides; WBC, white blood cells.

a Reference intervals from the LABOKLIN laboratory (Bad Kissingen, Germany).

b Significant differences (*P* ≤ 0.05) between study groups (Kruskal-Wallis-test with Bonferroni correction).

c Data were available for the indicated number of dogs.

d Reference ranges were given as a percentage of the total protein concentrations and were presented as g/l in the individual study groups.

Besides the 26 dogs included in SG II, 4 out of 23 dogs (17%) in SG I, 16 out of 17 (94%) in SG III, and none of the dogs in SG IV showed positive *Babesia* spp. ELISA antibody levels > 19 TE. The pathogen quantification in SG I revealed 840,000 to 102,000,000 *B. canis* pathogens/ml EDTA blood, with a median of 16,230,000 parasites. None of the SG II dogs (*Babesia* spp. antibody-positive) showed a positive PCR result. Out of the 17 dogs in SG III, 9 (52.9%) tested positive for *M. hematoparvum*, 5 (29.4%) for *M. hemocanis*, and 3 (17.6%) for both pathogens.

### 3.2 Hematological and biochemical results including CRP and ApoA-1

**Fig. 2:**
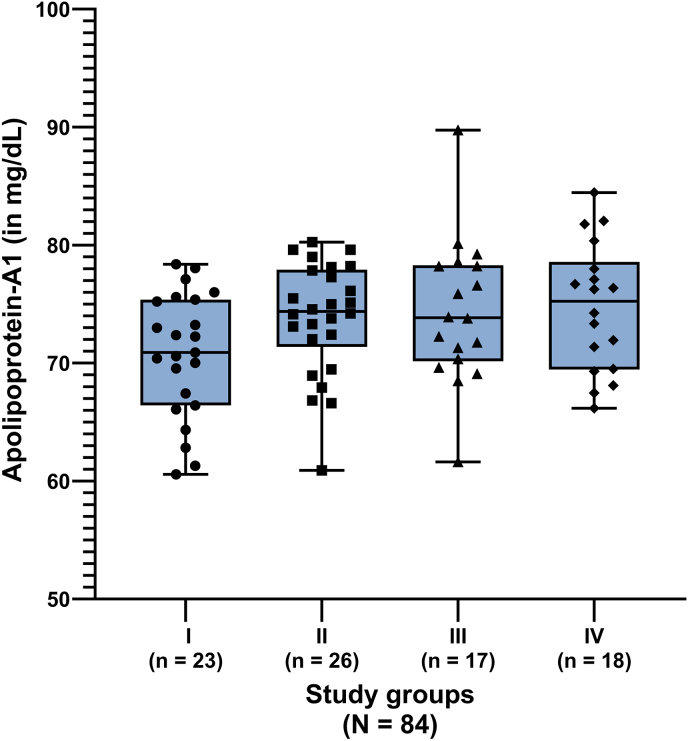
Apolipoprotein A-1 concentrations in 23 dogs with acute *Babesia canis* infections (I), 26 dogs tested serologically *Babesia* spp.-positive (II), dogs with acute hemoplasmosis (III) and clinically healthy dogs (IV).

Statistically significant differences between the SGs were observed for the hematological parameters hematocrit, white blood cell count, segmented neutrophils, lymphocytes, eosinophils, and platelet count, as well as for the biochemical parameters total protein, albumin, triglycerides, and CRP (Table 2). Globulin concentrations differed significantly between SGs I-III and III-IV (Table 2). In serum protein electrophoresis, statistically significant differences in concentrations of beta globulins (SG I-II) and gamma globulins (SG I-II and SG I-III) were noted (Table 2). The values of monocytes, cholesterol, and ApoA-1 concentrations did not differ significantly (Table 2, Fig. 2).

When comparing dogs with fever to dogs without fever using Mann-Whitney U-test, no statistical significance was found in ApoA-1 concentrations (*P* = 0.18), monocytes (*P* = 0.15), alpha globulins (*P* = 0.42), and beta globulins (*P* = 0.61). Statistical significance was demonstrated for hematocrit, platelet count, white blood cell count, segmented neutrophils, lymphocytes, eosinophils, total protein, albumin, CRP, and gamma globulins (*P* < 0.001 each), as well as triglycerides (*P* = 0.02) and cholesterol (*P* = 0.005) (Table 3).

**Table 3:** Rectal temperature, hematological and biochemical results, serum protein electrophoresis results, and apolipoprotein A-1 concentrations in dogs with (> 39.0 °C) and without fever.

Parameter	Dogs with fever (*n* = 18)		Dogs without fever (*n* = 66)		RI^a^	*P*
	Median (Range)	IQR	Median (Range)	IQR		
RT (°C)	39.5 (39.1–40.2)	39.2–39.9	38.3 (37.3–39.0)	38.2–38.5	≤ 39.0	<0.001
HCT (%)	35.5 (19.0–57.0)	31.0–46.3	50.0 (20.0–59.0)	46.0–53.8	44.0–52.0	<0.001
WBC (×10^9^/l)	4.4 (1.1–13.3)	3.2–6.4	8.8 (1.1–24.0)	7.5–10.8	6.0–12.0	<0.001
Seg (×10^9^/l)	2.9 (0.9–8.6)	2.4–4.3	4.8 (0.7–13.1)	4.1–6.1	3.0–9.0	<0.001
Lym (×10^9^/l)	0.6 (0.1–2.8)	0.3–1.5	2.5 (0.3–4.9)	1.8–3.4	1.0–3.6	<0.001
Eos (×10^9^/l)	0.0 (0.0–1.4)	0.0–0.1	0.6 (0.0–8.0)	0.3–0.9	0.04–0.6	<0.001
Mono (×10^9^/l)	0.4 (0.1–3.4)	0.3–0.8	0.6 (0.0–2.0)	0.4–0.8	0.04–0.5	0.15
PLT (×10^9^/l)	31.5 (11–236)	17–88	201 (23–420)	160–240	150–500	<0.001
TP (g/l)	56.0 (43.4–69.5)	48.8–59.4	62.3 (37.9–76.9)	57.4–65.3	54.0–57.0	<0.001
Alb (g/l)	31.5 (25.7–41.6)	27.5–35.0	37.2 (20.7–42.6)	35.0–39.5	25.0–44.0	<0.001
Glob (g/l)	23.0 (14.8–34.5)	18.5–27.9	24.7 (13.6–42.4)	21.6–28.0	< 45.0	0.25
Chol (mmol/l)	7.2 (3.9–10.3)	5.0–8.2	5.4 (2.9–9.8)	4.5–6.5	3.1–10.1	0.005
Trigly (mmol/l)	1.1 (0.5–1.6)	0.7–1.3	0.7 (0.3–4.6)	0.5–1.0	< 3.9	0.02
CRP (mg/l)	138.6 (0.3–292.5)	60.8–214.4	1.5 (0.1–152.0)	0.7–4.0	< 15.0	<0.001
Alb/Glob	1.5 (0.8–2.2)	1.0–1.4	1.5 (0.8–2.5)	1.0–1.4	> 0.59	0.65
EAlb (g/l)	30.0 (23.0–38.9)	25.7–32.6 (*n* = 17)^c^	33.4 (14.2–42.9)	29.2–36.8	44–66%^b^	0.04
Ealpha (g/l)	7.8 (5.0–12.7)	6.7–8.9 (*n* = 17)^c^	8.2 (4.7–41.5)	7.0–9.6	9–24%^b^	0.42
Ebeta (g/l)	9.9 (6.6–18.7)	8.4–14.0 (*n* = 17)^c^	10.9 (3.5–25.4)	8.7–13.3	9–32%^b^	0.61
Egamma (g/l)	4.9 (3.3–10.1)	4.1–5.1 (*n* = 17)^c^	8.6 (2.7–20.3)	6.0–10.0	5–20%^b^	<0.001
ApoA-1 (mg/dl)	71.6 (62.8–78.4)	69.3–75.7	74.1 (60.6–89.7)	69.5–78.0	–	0.18

*Abbreviations*: Alb, albumin; ApoA-1, apolipoprotein A-1; Chol, cholesterol; CRP, c-reactive protein; Eos, eosinophils; EAlb, albumin-section serum protein electrophoresis; Ealpha, alpha-globulin-section serum protein electrophoresis; Ebeta, beta-globulin-section serum protein electrophoresis; Egamma, gamma-globulin-section serum protein electrophoresis; Glob, globulin; HCT, hematocrit; IQR, interquartile range; Lym, lymphocytes; PLT, platelets; Mono, monocytes; RI, reference interval; Seg, segmented neutrophils; RT, rectal temperature; TP, total protein; Trigly, triglycerides; WBC, white blood cells.

a Reference intervals from the LABOKLIN laboratory (Bad Kissingen, Germany).

b Reference ranges were given as a percentage of the total protein concentrations, and were presented as g/l in the individual study groups.

c Data regarding electrophoresis results were available for 17 out of 18 dogs (94%).

Results of the correlation analysis, including hematology and biochemistry results, CRP, and ApoA-1 concentrations, as well as *Babesia* spp. antibody levels are listed in Supplementary Table S1. ApoA-1 concentrations showed a moderate negative correlation with the cholesterol concentration (*ρ* = −0.329, *P* = 0.002), and a moderate positive correlation with the number of eosinophils (*ρ* = 0.364, *P* = 0.001). CRP revealed strong negative correlations with hematocrit (*ρ* = −0.565), platelet (*ρ* = −0.624), lymphocyte (*ρ* = −0.580) and eosinophil (*ρ* = −0.538) counts, and albumin concentration (*ρ* = −0.691) (*P* < 0.001 each). The CRP concentrations were moderately positively correlated with rectal temperature (*ρ* = 0.310, *P* = 0.005) and triglyceride concentration (*ρ* = 0.413, *P* < 0.001). Moderate negative correlations with the white blood cell count (*ρ* = −0.392, *P* < 0.001), total protein concentration (*ρ* = −0.483, *P* = < 0.001), the albumin/globulin-ratio (*ρ* = −0.395, *P* < 0.001), gamma globulin concentration (*ρ* = −0.306, *P* = 0.005), and *Babesia* spp. antibody levels (*ρ* = −0.478, *P* < 0.001) were additionally detected (Supplementary Table S1). In dogs with acute *B. canis* infection (SG I), no significant correlation was demonstrated for pathogen quantification and ApoA-1 concentrations (*ρ* = −0.391, *P* = 0.07).

## 4 Discussion

In the present study, significant higher CRP concentrations as a suitable marker for characterization of APRs were demonstrated in dogs with acute *B. canis* infections. However, no statistical significance of ApoA-1 concentrations could be demonstrated in dogs with acute *B. canis* infections. Similarly, no significant changes in ApoA-1 concentrations were detected in dogs with acute hemotrophic *Mycoplasma* infections, in dogs tested serologically positive for *Babesia* spp., and in clinically healthy dogs.

By comparing dogs with fever to dogs without fever, significant differences were observed in CRP, triglyceride, and cholesterol concentrations, but not in ApoA-1 concentrations. The diagnostic value of ApoA-1 in dogs with fever, APRs, and dogs with acute *B. canis* and hemotrophic *Mycoplasma* infections, respectively, seems questionable. Whether there is an immunological impact in dogs serologically positive for *Babesia* spp. and those with acute hemotrophic *Mycoplasma* infections needs to be further evaluated, but no evidence of significant APR was observed in the present study in both study groups. Plasma lipoproteins can directly modulate the host response in inflammatory diseases. ApoA-1 inactivates bacterial endotoxin and inhibits LPS-induced production of inflammatory cytokines from monocytes (Giunti et al., 2020). In acute inflammatory infectious and septic diseases in humans, circulating ApoA-1 concentrations decrease (Catapano et al., 2014). A decrease in serum ApoA-1 concentrations was associated with higher illness severity, lethality, and susceptibility to infection in critical patients in human medicine (Gordon et al., 2001). In contrast, ApoA-1 seemed to be no valuable marker for APRs nor valuable as a negative acute-phase protein in canine babesiosis and hemoplasmosis in our study. To the best of our knowledge, no information regarding differences in lipoprotein mechanisms in dogs and humans potentially affecting ApoA-1 concentrations in acute infectious diseases is available at the time of our study.

In contrast to the findings in our study, ApoA-1 may also act as a negative acute-phase protein based on a negative correlation with CRP in one study (Escribano et al., 2016); however such negative correlation of ApoA-1 concentrations with CRP were neither detected in two previous studies in septic dogs (Giunti et al., 2020; Bulgarelli et al., 2023).

However, relatively low numbers of dogs were included in previous studies, and therefore, statistical significance might have been lost. Although not statistically significant when comparing the different SGs in the present study, lower ApoA-1 concentrations were observed in dogs with acute *B. canis* infection, like in dogs with leishmaniasis (Escribano et al., 2016). By contrast, statistically significantly higher ApoA-1 concentrations in dogs with canine babesiosis compared to healthy ones were detected in another study (Milanovic et al., 2019). However, a strong APR was demonstrated in dogs with acute *B. canis* infection, represented by significantly higher CRP in our study, and SAA concentrations in the previously mentioned study (Milanovic et al., 2019). Both studies also concurred on the hematological findings of anemia, leukopenia, thrombocytopenia, and lymphopenia in dogs with acute *B. canis* infection, with statistically significant differences compared to healthy dogs. All hematological findings are described as typical for acute *B. canis* infections (Seibert et al., 2022; Weingart et al., 2023; Eisenecker et al., under review).

The divergent results of ApoA-1 concentrations in dogs with acute *B. canis* infection may be linked to the use of different diagnostic assays, originally designed for the quantification of ApoA-1 in humans. In this study, an immunoturbidimetric assay with photometric determination of an antigen-antibody reaction between antibodies against ApoA-1 and the concentration of ApoA-1 in the sample was used, whereas a radioimmunoassay was included in the other study (Milanovic et al., 2019). Furthermore, different genotypes of *B. canis* may impact the severity of clinical signs as well as hematological and biochemical parameters, with the most pathogenic genotypes circulating in eastern Europe (Carcy et al., 2015), which may be true for the Serbian dogs from Belgrade in the other study (Milanovic et al., 2019). The situation regarding *B. canis* genotypes in Germany is not well described yet; however, a bright heterogenicity of genotypes has been described in German dogs without stays abroad based on the *B. canis-*specific *Bc28.1* gene (Helm et al., 2022). The severity of hematological findings and CRP concentrations is significantly influenced by the number of *B. canis* parasites circulating in the peripheral blood (Von Hohnhorst et al., 2025). High numbers of *B. canis* specimens were detected in SG I with a median of 16,230,000/ml EDTA blood in our study, which most likely has affected the APR reflected by CRP concentrations. ApoA-1 concentrations did not significantly correlate with the number of *B. canis* parasites in dogs in SG I but showed a moderate negative correlation with the cholesterol concentration. No further moderate or strong correlations with any other parameter were observed, while another study detected a positive correlation between ApoA-1 and SAA (Milanovic et al., 2019).

In dogs with septic peritonitis, significantly lower ApoA-1 concentrations were detected compared to healthy control dogs, while this could not be demonstrated in cases of parvoviral enteritis, pyometra, and miscellaneous conditions with no statistically significant CRP and albumin values in the examined study groups (Giunti et al., 2020). Further studies are needed to prove the value of ApoA-1 concentrations as a marker for APR in dogs with acute *B. canis* infection.

In general, lower concentrations of HDL-cholesterol were observed in dogs with babesiosis (Mrljak et al., 2014; Rossi et al., 2014), as well as in cattle infected with *Babesia bovis* (Goodger et al., 1981) and humans infected with *Babesia* species (Cunha et al., 2000). However, increased cortisol and insulin levels were detected in dogs with babesiosis (Matijatko et al., 2014; Zygner et al., 2015), with hypercholesteremia to be expected. In our study, no significant differences in cholesterol concentrations were observed between the different study groups, so the impact of canine babesiosis remains unclear.

Elevated serum triglyceride concentrations with hypertriglyceridemia were detected in hamsters after administration of lipopolysaccharides (LPS) (Feingold et al., 1993). In the present study, triglyceride concentrations were significantly higher in dogs with acute *B. canis* infection than in the other SGs. Serum triglyceride concentrations were also increased in another study after treatment of *B. canis* infections, which suggested the involvement of this lipid class in the APR (Mrljak et al., 2014). This is in accordance with the present study and may be explained by triglycerides as a source of fatty acids and as a part of the innate defense mechanism to neutralize lipophilic toxins (Khovidhunkit et al., 2004).

It was not surprising that neither APRs nor any significant difference in CRP concentrations were demonstrated in dogs tested serologically positive and clinically healthy dogs. The low concentrations of CRP and absence of APR in serologically positive dogs can be explained by previous pathogen contact in the past, leading to an increase in antibody levels. In a recent study, the protective nature of positive antibody levels could be demonstrated in dogs with acute *B. canis* infections (Von Hohnhorst et al., 2025). This study detected a negative correlation between antibody levels and the severity of hematological abnormalities, CRP concentrations, and pathogen quantification in dogs with acute *B. canis* infection (Von Hohnhorst et al., 2025). In the present study, no significant difference was observed for any of the parameters tested in serologically positive dogs or those infected with hemotrophic *Mycoplasma*. In consequence, no evidence for APR involvement could be demonstrated in these two study groups.

Hemotrophic *Mycoplasma* infections might not cause a significant APR in most cases (Messick, 2004). If clinical signs and/or hemolytic anemia are detected, dogs are usually splenectomized and/or immunocompromised (Tasker, 2022), resulting in a chronic carrier status after clinical signs resolved (Messick, 2004). In the dogs included in our study, no underlying comorbidities were reported by the veterinarians, and no corticosteroids were applied, all of which might have potentially caused immunosuppression. Therefore, the absence of APRs seemed not surprising on dogs with hemotrophic *Mycoplasma* infections. However, the pathogenesis of hemotrophic *Mycoplasma* infections in dogs is still poorly understood, including the significance of APRs.

A limitation of the study is that the time frame between the first presentation of dogs with acute babesiosis and the initial *B. canis* infection was unknown and may impact observed lipoprotein changes. Also, potential comorbidities could not be ruled out for sure; however, other vector-borne diseases such as ehrlichiosis and leishmaniosis were ruled out as far as possible. Cross-reactions with other protozoan agents such as *Leishmania infantum*, *Babesia gibsoni*, and *Babesia vogeli* are known to occur in ELISA testing. However, cross-reactions usually cause questionable or low positive antibody levels, so high levels > 60 TE were chosen as inclusion criteria in SG II.

## 5 Conclusions

The value of ApoA-1 as an acute-phase protein in dogs with acute *B. canis* infection is questionable. Generally, higher triglyceride and cholesterol concentrations were observed in dogs with fever. However, when comparing dogs with acute *B. canis* infections to the other SGs, statistical significance could only be demonstrated for triglycerides, but not for cholesterol. CRP is a positive acute-phase protein as well as a suitable marker for characterization of APRs and was correlated with clinical as well as laboratory parameters in this study. However, no significant correlation of CRP with ApoA-1 concentrations could be observed, neither with clinical nor laboratory parameters. Whether there is an immunological impact in dogs tested serologically positive for *B. canis* and those with acute hemotrophic *Mycoplasma* infections needs to be further evaluated, but no evidence of significant APR was observed in the present study.

## Ethical approval

Not applicable.

## CRediT authorship contribution statement

**Imke M. von Hohnhorst:** Investigation, Visualization, Writing - original draft. **Andreas Moritz:** Supervision, Writing - review & editing. **Clara M. Eisenecker:** Investigation, Writing - review & editing. **Christina Strube:** Writing - review & editing. **Torsten J. Naucke:** Writing - review & editing. **Elisabeth Müller:** Writing - review & editing. **Ingo Schäfer:** Conceptualization, Supervision, Writing - review & editing.

## Funding

This research did not receive any specific grant from funding agencies in the public, commercial, or not-for-profit sectors.

## Declaration of competing interests

The authors declare the following financial interests/personal relationships which may be considered as potential competing interests: Torsten J. Naucke and Ingo Schäfer are employees, and Elisabeth Müller is the CEO of Laboklin GmbH & Co. KG; Andreas Moritz, Christina Strube, and Ingo Schäfer have repeatedly lectured for and/or acted as consultants for diagnostic and (veterinary) pharmaceutical companies; Andreas Moritz and Christina Strube have previous and ongoing research collaborations with various diagnostic and (veterinary) pharmaceutical companies. The other authors declare that they have no known competing financial interests or personal relationships that could have appeared to influence the work reported in this paper.

## Data Availability

The data supporting the conclusions of this article are included in this published article and its supplementary file.

## Appendix A Supplementary data

Supplementary file 1 Spearman correlation analysis to determine potential correlations between hematological results, triglycerides, cholesterol and CRP concentrations, *Babesia* spp. antibody levels, and results of serum protein electrophoresis. Supplementary file 1

## References

[bib1] Barker E.N., Tasker S., Day M.J., Warman S.M., Woolley K., Birtles R. (2010). Development and use of real-time PCR to detect and quantify *Mycoplasma haemocanis* and “*Candidatus* Mycoplasma haematoparvum” in dogs. Vet. Microbiol..

[bib2] Bermudes A.C.G., de Carvalho W.B., Zamberlan P., Muramoto G., Maranhao R.C., Delgado A.F. (2018). Changes in lipid metabolism in pediatric patients with severe sepsis and septic shock. Nutrition.

[bib3] Bulgarelli C., Ciuffoli E., Troia R., Goggs R., Dondi F., Giunti M. (2023). Apolipoprotein A1 and serum amyloid A in dogs with sepsis and septic shock. Front. Vet. Sci..

[bib4] Carcy B., Randazzo S., Depoix D., Adaszek L., Cardoso L., Baneth G. (2015). Classification of *Babesia canis* strains in Europe based on polymorphism of the *Bc28.1*-gene from the *Babesia canis Bc28* multigene family. Vet. Parasitol..

[bib5] Catapano A.L., Pirillo A., Bonacina F., Norata G.D. (2014). HDL in innate and adaptive immunity. Cardiovasc. Res..

[bib6] Chien J.Y., Jerng J.S., Yu C.J., Yang P.C. (2005). Low serum level of high-density lipoprotein cholesterol is a poor prognostic factor for severe sepsis. Crit. Care Med..

[bib31] Cohen J. (1988). Statistical Power Analysis for the Behavioral Sciences.

[bib8] Cunha B.A., Crean J., Rosenbaum G. (2000). Lipid abnormalities in babesiosis. Am. J. Med..

[bib9] Escribano D., Tvarijonaviciute A., Kocaturk M., Ceron J.J., Pardo-Marin L., Torrecillas A. (2016). Serum apolipoprotein-A1 as a possible biomarker for monitoring treatment of canine leishmaniosis. Comp. Immunol. Microbiol. Infect. Dis..

[bib10] Feingold K.R., Hardardottir I., Memon R., Krul E.J., Moser A.H., Taylor J.M., Grunfeld C. (1993). Effect of endotoxin on cholesterol biosynthesis and distribution in serum lipoproteins in Syrian hamsters. J. Lipid Res..

[bib11] Giunti M., Grossi G., Troia R., Fracassi F., Dondi F. (2020). Evaluation of serum Apolipoprotein A1 in canine sepsis. Front. Vet. Sci..

[bib12] Giunti M., Troia R., Bergamini P.F., Dondi F. (2015). Prospective evaluation of the acute patient physiologic and laboratory evaluation score and an extended clinicopathological profile in dogs with systemic inflammatory response syndrome. J. Vet. Emerg. Crit. Care (San Antonio).

[bib13] Goddard A., Wiinberg B., Schoeman J.P., Kristensen A.T., Kjelgaard-Hansen M. (2013). Mortality in virulent canine babesiosis is associated with a consumptive coagulopathy. Vet. J..

[bib14] Goodger B.V., Wright I.G., Mahoney D.F. (1981). *Babesia bovis* (Argentina): studies of plasma lipids and lipoproteins during acute infections in cattle. Z. Parasitenkd..

[bib15] Gordon B.R., Parker T.S., Levine D.M., Saal S.D., Wang J.C., Sloan B.J. (2001). Relationship of hypolipidemia to cytokine concentrations and outcomes in critically ill surgical patients. Crit. Care Med..

[bib16] Helm C., Weingart C., Ramünke S., Schäfer I., Müller E., Samson-Himmelstjerna G.V. (2022). High genetic diversity of *Babesia canis* (Piana & Galli-1 Valerio, 1895) in a recent local outbreak in Berlin/Brandenburg, Germany. Transbound. Emerg. Dis..

[bib17] Khovidhunkit W., Kim M.S., Memon R.A., Shigenaga J.K., Moser A.H., Feingold K.R., Grunfeld C. (2004). Effects of infection and inflammation on lipid and lipoprotein metabolism: mechanisms and consequences to the host. J. Lipid Res..

[bib18] Matijatko V., Torti M., Kis I., Iva S., Stokovic I., Vranjes-Duric S. (2014). Serum cortisol and insulin concentrations in dogs naturally infected with *Babesia canis*. Vet. Arh..

[bib19] Messick J.B. (2004). Hemotrophic mycoplasmas (hemoplasmas): a review and new insights into pathogenic potential. Vet. Clin. Pathol..

[bib20] Milanovic Z., Vekic J., Radonjic V., Ilic Bozovic A., Zeljkovic A., Janac J. (2019). Association of acute *Babesia canis* infection and serum lipid, lipoprotein, and apoprotein concentrations in dogs. J. Vet. Intern. Med..

[bib21] Mrljak V., Kucer N., Kules J., Tvarijonaviciute A., Brkljacic M., Crnogaj M. (2014). Serum concentrations of eicosanoids and lipids in dogs naturally infected with *Babesia canis*. Vet. Parasitol..

[bib22] Olmeda A.S., Armstrong P.M., Rosenthal B.M., Valladares B., del Castillo A., de Armasc F. (1997). A subtropical case of human babesiosis. Acta Trop..

[bib23] Rossi G., Kules J., Rafaj R.B., Mrljak V., Lauzi S., Giordano A., Paltrinieri S. (2014). Relationship between paraoxonase 1 activity and high density lipoprotein concentration during naturally occurring babesiosis in dogs. Res. Vet. Sci..

[bib24] Seibert S., Rohrberg A., Stockinger A., Schaalo S., Marz I. (2022). [Occurrence of canine babesiosis in dogs in the Rhine-Main area of Hesse, Germany - a case study of 81 dogs]. Tierarztl. Prax. Ausg. K Kleintiere Heimtiere.

[bib25] Tanaka S., Couret D., Tran-Dinh A., Duranteau J., Montravers P., Schwendeman A., Meilhac O. (2020). High-density lipoproteins during sepsis: from bench to bedside. Crit. Care.

[bib26] Tasker S. (2022). Hemotropic *Mycoplasma*. Vet. Clin. North Am. Small Anim. Pract..

[bib27] Turna H., Vichova B., Miterpakova M., Szarkova A., Baneth G., Svoboda M. (2022). Correction: clinical and hematologic findings in *Babesia canis* infection in eastern Slovakia. Acta Parasitol..

[bib28] Von Hohnhorst I.M., Moritz A., Eisenecker C.M., Strube C., Rodjana K., Müller E., Schäfer I. (2025). Impact of pathogen quantification, antibody levels, and stays abroad on hematological as well as biochemistry parameters and acute-phase proteins in dogs with acute *Babesia canis* infections in Germany. Parasites Vectors.

[bib29] Weingart C., Helm C.S., Müller E., Schäfer I., Skrodzki M., von Samson-Himmelstjerna G. (2023). Autochthonous *Babesia canis* infections in 49 dogs in Germany. J. Vet. Intern. Med..

[bib30] Zygner W., Gojska-Zygner O., Baska P., Dlugosz E. (2015). Low T3 syndrome in canine babesiosis associated with increased serum IL-6 concentration and azotaemia. Vet. Parasitol..

